# An enhanced Swin Transformer for soccer player reidentification

**DOI:** 10.1038/s41598-024-51767-4

**Published:** 2024-01-11

**Authors:** Sara Akan, Songül Varlı, Mohammad Alfrad Nobel Bhuiyan

**Affiliations:** 1 Department of Computer Engineering, Yildiz Technical University, Istanbul, Turkey; 2 Department of Medicine, Louisiana State University Health, Shreveport, LA USA

**Keywords:** Engineering, Computer science

## Abstract

The re-identification (ReID) of objects in images is a widely studied topic in computer vision, with significant relevance to various applications. The ReID of players in broadcast videos of team sports is the focus of this study. We specifically focus on identifying the same player in images taken at any given moment during a game from various camera angles. This work varies from other person ReID apps since the same team wears very similar clothes, there are few samples for each identification, and image resolutions are low. One of the hardest parts of object ReID is robust feature representation extraction. Despite the great success of current convolutional neural network-based (CNN) methods, most studies only consider learning representations from images, neglecting long-range dependency. Transformer-based model studies are increasing and yielding encouraging results. Transformers still have trouble extracting features from small objects and visual cues. To address these issues, we enhanced the Swin Transformer with the levering of CNNs. We created a regional feature extraction Swin Transformer (RFES) backbone to increase local feature extraction and small-scale object feature extraction. We also use three loss functions to handle imbalanced data and highlight challenging situations. Re-ranking with k-reciprocal encoding was used in this study's retrieval phase, and its assessment findings were provided. Finally, we conducted experiments on the Market-1501 and SoccerNet-v3 ReID datasets. Experimental results show that the proposed re-ID method reaches rank-1 accuracy of 96.2% with mAP: 89.1 and rank-1 accuracy of 84.1% with mAP: 86.7 on the Market-1501 and SoccerNet-v3 datasets, respectively, outperforming the state-of-the-art approaches.

## Introduction

Recently, automated sports video analysis has attracted considerable interest, especially in team sports like soccer, basketball, and volleyball, due to the increasing demand for semantic information extraction from sports experts and fans. The outcomes of sports analysis can be implemented in a variety of scenarios, such as TV storytelling, altering the training schedule, creating game statistics, and identifying the strengths and weaknesses of a team or a player. player re-identification is a cornerstone of modern sports analysis, enriching our understanding of the game, enhancing coaching decisions, and delivering a more comprehensive and engaging experience for fans and stakeholders alike. As a result, player ReID is a crucial research topic for maximizing the advantages of automatic sports analysis. It is necessary to connect the correct player to each track and link his/her actions and statistics to it to re-identify a player.

Reidentifying players in broadcast sports videos can be challenging due to factors like low video resolution, camera movement, player pose, lighting conditions, and uniform similarities among players. Players can be reidentified on the field using their faces and jersey numbers^1,2^. When the player's face is clearly visible in close-up shots, using face recognition as a ReID technique is effective, but it becomes impractical in overview shots. On the other hand, strategies based on jersey numbers have promise because they make up a sizable portion of a player's back uniform and because HD sports videos are growing in popularity. However, several external factors, like clocks, advertising logos, banners, player tilting, motion blur, and viewing angles, might potentially pose difficulties in accurately discerning jersey numbers^3^.

**Figure 1:**
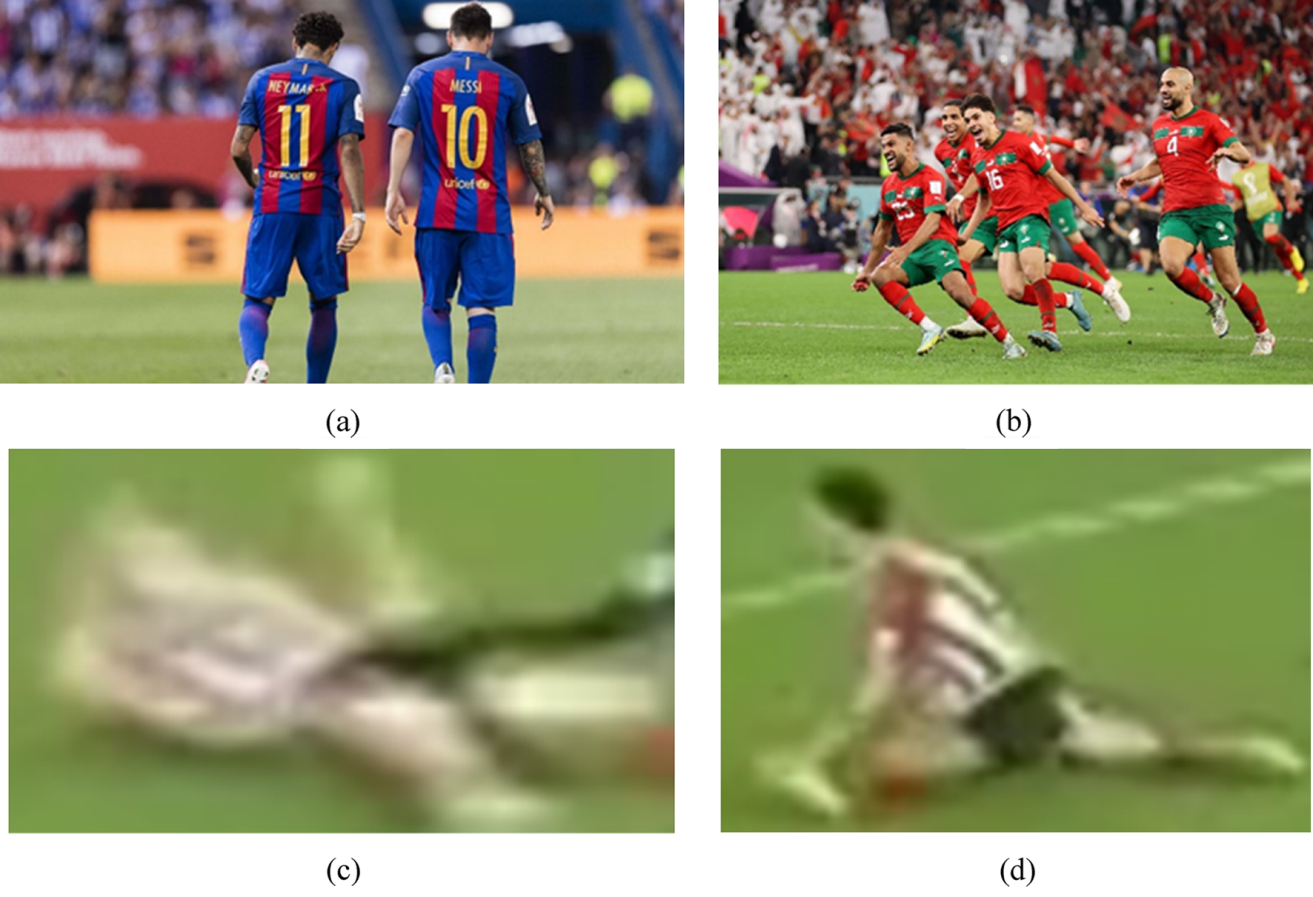
Some examples of various challenges: (**a**) Similar uniforms; (**b**) Occlusion; (**c**, **d**) low resolution and different body movements.

Soccer player ReID research is crucial for sports as a sub-branch of person ReID, but there is a lack of research on it thus far. In general, prior person ReID techniques gather attributes from whole images and then match gallery candidates based on how those attributes are represented visually. To create an effective representation, prior methods directly used global person features^4,5^. Object ReID tasks have been dominated by CNN-based techniques that are robust and discriminative in feature extraction^6–13^. The analysis of CNN-based techniques in the field of object ReID primarily concentrated on visual features. This paper focuses on the application of ReID technology in the context of soccer players. To be more specific, we want to develop a system that can accurately re-identify players from different camera angles at any moment throughout a game. This system has numerous potential applications, including player tracking across multiple cameras, automated highlight videos for a single player, and improving referee assistance tools^14–17^. The following significant differences exist between sport player ReID task and person ReID applications: (1) Players from the same team often look alike, as they wear the same team jerseys; (2) The quality of images in sport player ReID is often low, with occlusions and fast player movement making it harder to re-identify players. These problems are illustrated in Fig. 1. (3) Training machine learning models for sport player ReID is more challenging due to the limited number of samples for each identity.

This study proposes a technique known as RFES-ReID to address the challenging task of soccer player ReID. Swin Transformer serves as the backbone network in our study, and we take use of its local attention mechanism and superior large-range dependency modeling capability to enhance ReID's effectiveness. Furthermore, we designed a regional feature extraction block to take advantage of capturing fine-grained details from specific areas of interest within an image. The player ReID training process is then enhanced using a fusion loss function. Re-ranking is additionally used with the baseline model presented here to further improve performance and handle false matches. The main contributions of this paper are briefly summarized as follows.

- Using Swin Transformer as a backbone network to extract image features to address the issue of CNN's large-range dependency modeling and the high computational cost of traditional Transformers.
- The proposed RFES enhances feature extraction accuracy from small-scale objects and improves the model's local perception abilities by incorporating the benefits of CNNs.
- The soccer player ReID network is enhanced through the use of cross-entropy loss, triplet loss, and focal loss for accurate classification, handling unbalanced data, and considering inter-sample similarities and difficult-to-separate samples.
- The RFES-ReID framework provides competitive results on person and soccer player ReID benchmarks, namely Market-1501^18^ and SoccerNet-v3^19^.

In summary, the Pros and Cons of the proposed method are as follows.

The incorporation of the Swin Transformer as a backbone network offers effective large-range dependency modeling, addressing a key limitation of CNNs in soccer player re-identification. The Regional Feature Enhancement Strategy (RFES) improves small-scale object perception, enhancing local perception abilities. Versatile loss functions, including cross-entropy, triplet, and focal loss, contribute to accurate classification and robust handling of diverse scenarios. Although Market-1501 and SoccerNet-v3 benchmark results are competitive, there may be disadvantages such as higher computational costs, more implementation complexity, reliance on benchmark datasets, and hyperparameter sensitivity.

## Related works

Since soccer player ReID is a branch of person ReID and there do not exist many studies for sports player ReID, more studies for person ReID are taken into consideration here. Person ReID is a process that involves identifying and matching the same person across multiple images captured by different cameras. The goal is to find images of the same person from a gallery of images taken by various cameras that do not overlap. The task has a wide variety of possible uses for public safety, particularly in smart monitoring systems. Person ReID is a challenging process since a person's look differs across various cameras. This is due to a multitude of issues, including illumination variances, occlusion, position changes, and backdrop clutter.

Before deep learning algorithms came along, early studies on human re-recognition mostly concentrated on enhancing similarity metrics and manually improving visual features. Deep learning techniques have revolutionized person ReID tasks by automatically extracting superior features from person images and learning better similarity metrics, making them increasingly prevalent in modern applications. Deep learning techniques, in contrast to conventional approaches, have the ability to automatically extract better features of person images and learn better similarity metrics at the same time. CNN-based approaches have consistently been at the forefront of the extraction of distinguishable and robust features, which play a vital role in the process of ReID^6,20,21^. Recent years have seen a significant improvement in the task of person ReID thanks to high-performance deep learning algorithms^22–25^. CNNs are used in the current methods to solve the person ReID problem using a wide range of techniques, including multi-class classification^26–28^, verification^29–31^, distance-based deep approaches^5,32,33^ and part-based deep approaches^6,34–37^. Although CNN approaches have achieved significant success^38^, they analyze one local area at a time and encounter a reduction in detailed information due to the use of convolution and downsampling operators such as pooling and stepwise convolution. CNN focuses on detecting edges, shapes, and distinctive features of a person, but it does not consider the interdependencies and interactions among all of these features. Consequently, when images of people are subjected to rotation or taken from various perspectives, the performance of the CNN model typically falls short of expectations. However, the development of the attention mechanism has effectively addressed the issue of information loss in convolutional neural networks^39^.

### Transformer based person ReID

Transformer^40^ is a popular model in natural language processing (NLP) and outperforms RNN-based and CNN-based models in machine translation tasks. Vision Transformer (ViT)^41^ inspired by Transformers' scaling in NLP, a standard Transformer was directly applied to images with minimal modifications. ViT was introduced in 2020 for image classification, and its application later expanded to various computer vision tasks beyond classification. This model outperforms CNNs in image classification tasks. The utilization of transformer models in computer vision, particularly in the domain of person ReID, is increasingly prevalent among researchers. CNN primarily emphasizes the extraction of edge, shape, and person features while neglecting to account for their interrelationships. The effectiveness of feature extraction for the recognition of images has been established by ViT and Data efficient image Transformers (DeiT), indicating the practicality of a CNN-based technique. Person re-identification using CNNs captures person features without considering their relationships, whereas the emergence of Vision Transformers effectively addresses this issue by using a multi-head attention mechanism and excelling in diverse scenarios like different body movements and occlusions. ViT and Data efficient image Transformers (DeiT)^42^ demonstrate that Transformers can serve as practical alternatives for feature extraction in computer vision tasks.

TransReID^43^ is a method for person ReID based on ViT by adding the jigsaw patch module (JPM) and the side information embeddings (SIE) but it requires a larger pre-training dataset due to the domain gap between ImageNet and ReID datasets. Luo et al.^44^ proposed TransReID-SSL aims to bridge this gap by examining self-supervised learning methods with ViT pretrained on unlabeled person images. The results show that ViT significantly outperforms ImageNet supervised pre-training models on ReID tasks.

The ViT model's patch size is fixed and scaled uniformly. The scale is uniform for the domain of NLP, while the patch size of the image in computer vision is variable and may be either large or small. In computer vision, the patch size often must be modified for downstream tasks like target recognition, pixel-level segmentation, etc. The presence of potential computing problems for ViT due to modifying patch sizes and its limited viability for downstream tasks if patches remain constant has been addressed by the emergence of the Swin Transformer^45^.

Swin Transformer is a sliding-window variant of ViT can effectively tackle this problem and improve performance in tasks like classification, detection, and segmentation. Many of the hyperparameters typically present in CNNs can be manually adjusted in Swin Transformer. These include the number of network blocks, the number of layers within each block, and the dimensions of the input image, among others. Several studies have employed a Swin Transformer for object ReID^39,46^. However, they used an additional segmentation step that is a crucial process that involves categorizing entire regions, requiring high computational requirements and processing times, and can lead to inaccuracies that impact subsequent classification tasks. It uses a hierarchical network structure like CNNs. It uses a shifted window mechanism to share pixel points in different windows by dividing ViT sample blocks into varying sizes based on hierarchy. Swin Transformer improves the network's "perceptual field" and information utilization compared to the TransReID and TransReID-SSL methods used for person ReID.

### Loss metrics for person re-identifying

In the design of deep metric learning for person ReID, many well-known loss functions are routinely utilized. These include identity loss, verification loss, and triplet loss. The person ReID is formulated as a classification problem by the identity loss. When given a query image, the ReID system returns the ID of the person who is the focus of the search. To determine identity loss, the cross entropy^47^ function is frequently used. The verification loss looks for the best pair-wise arrangement of two subjects. Contrastive loss^48^ and binary verification loss^29^ are two other frequently used functions. The contrastive loss can be represented with a linear combination of a pairwise distance in embedding feature space and a binary label, while binary verification loss distinguishes between positive and negative image pair sets. The approach known as triplet loss^49^, considers ReID as a clustering task, according to the principle of regulating the feature distance between positive and negative pairings. To be more specific, there should be a defined margin within which the positive pair is separated from the negative pair. We notice that the majority of methods combine the aforementioned three types of loss^50,51^. However, the issue of data imbalance has only been considered in a few methods^52,53^. Focal loss has been shown to be efficient for networks that prioritize learning hard-to-separate examples during training in order to tackle the issue of data imbalance^54^. The overall performance of a person ReID can be improved by including the focal loss in deep metric learning. This prompted us to use a fusion loss function that incorporates not only cross-entropy and Triplet loss but also focal loss.

### Proposed method

**Figure 2:**
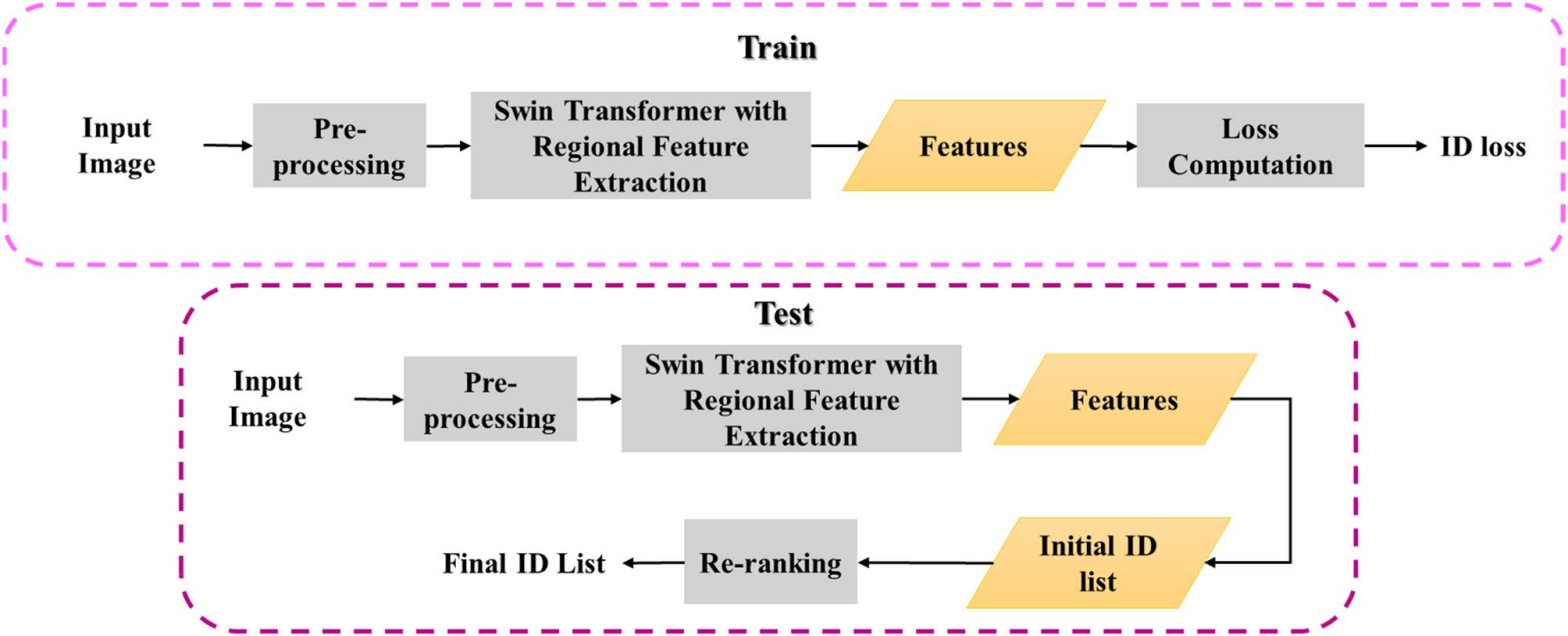
General RFES-ReID method block diagram.

The proposed framework pre-processes and feeds the input query image into the RFES-ReID module. A fusion loss module is then added to the training procedure to get ID loss. On the other hand, the procedure in the inference mode is precisely the same, with the exception that a re-ranking optimization is used after the generation of the initial ID list. Figure 2 shows the framework of the proposed method.

### Regional feature extraction Swin Transformer module (RFES)

**Figure 3:**
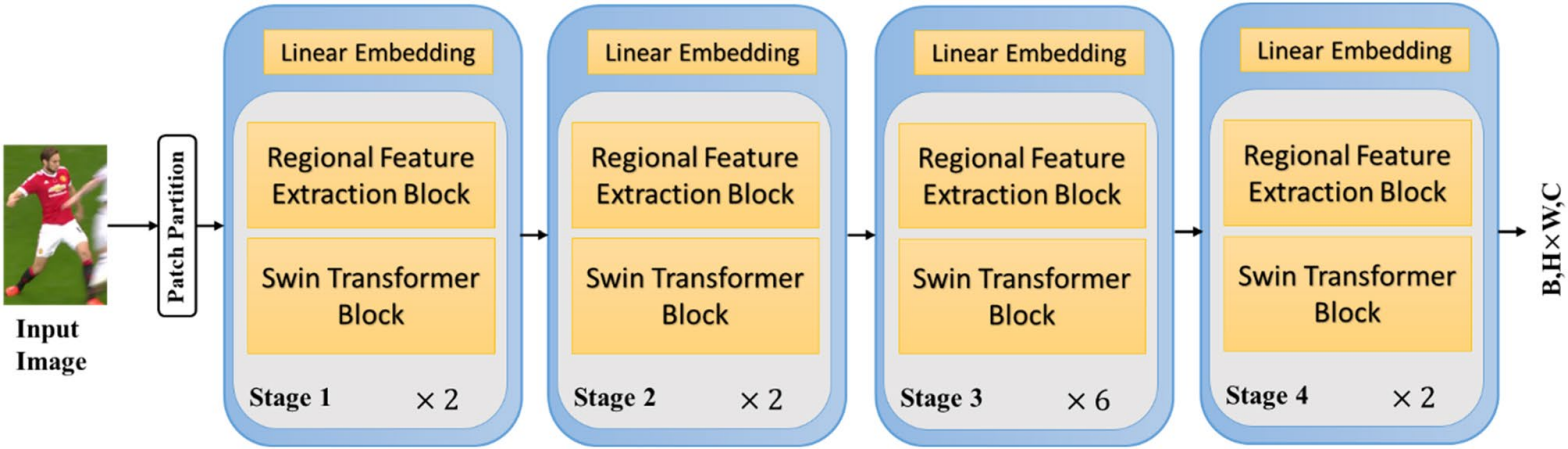
Regional feature extraction Swim transformer architecture.

There are four different types of the Swin Transformer: Swin-T, Swin-S, Swin-B, and Swin-L^45^. This study use Swin-T, which takes into consideration the uniqueness and computational difficulty of person ReID images. Respectively, there are 2, 2, 6, and 2 blocks on each stage. The flowchart of the network's regional feature extraction Swin Transformer (RFES) is shown in Fig. 3.

#### A quick review of Swin Transformer

**Figure 4:**
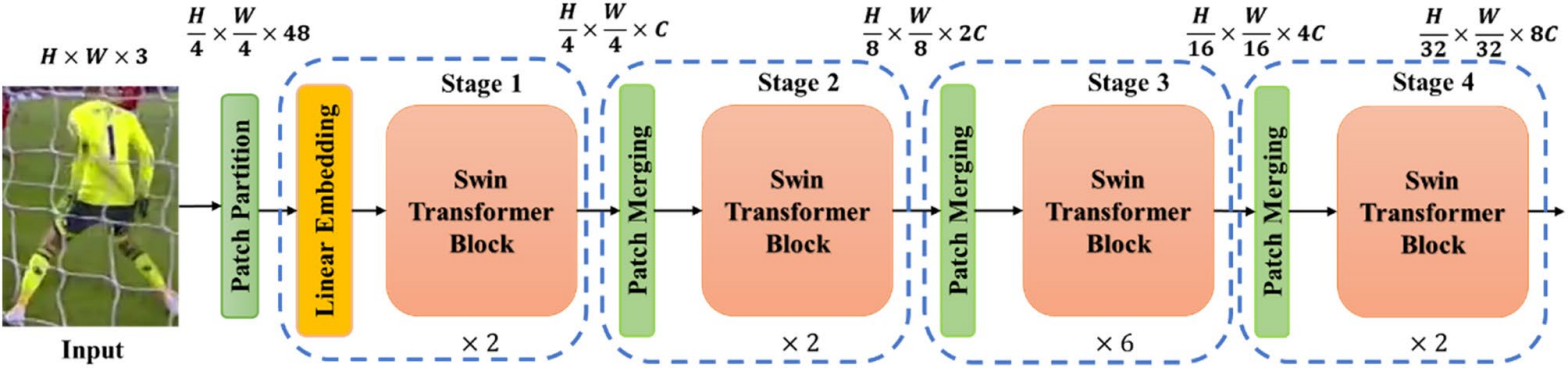
The Architecture of the Swin Transformer Model.

As mentioned before, the Swin Transformer is a ViT-based module that uses sliding windows. The architecture of the Swin Transformer is shown in Fig. 4. It replaces the Multi-Head Attention mechanism (MSA) with W-MSA and SW-MAS, enhancing computational efficiency and classification accuracy through the use of restricted and sliding windows.

**Figure 5:**
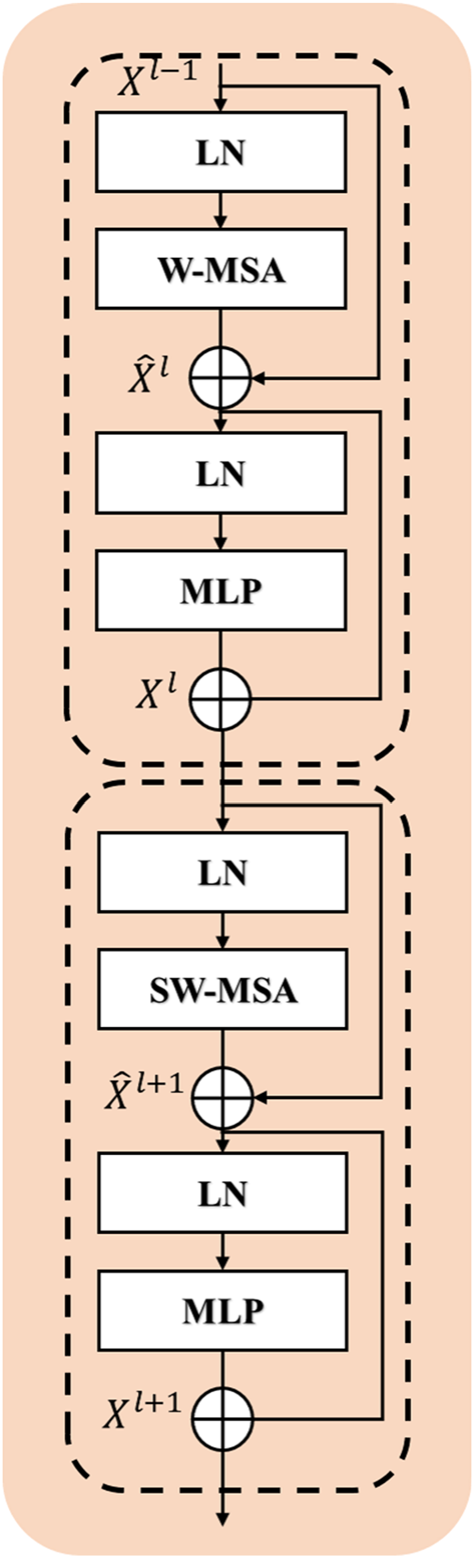
Blocks of Swin Transformers.

Swin Transformer, as seen in Fig. 5, first reshapes an input of size $H\times W\times C$ into a $\frac{HW}{{M}^{2}}\times {M}^{2}\times C$ feature by dividing the input into non-overlapping $M\times M$ local windows, where $\frac{HW}{{M}^{2}}$ is the total number of windows. The standard self-attention (also known as local attention) is then calculated independently for each window. The query, key, and value matrices (*Q*, *K* and *V*) are calculated for a local window feature $X\in {\mathbb{R}}^{{M}^{2}\times C}$ as:

1 $$Q=X{P}_{Q},K=X{P}_{K},V=X{P}_{V},$$

where ${P}_{Q},{P}_{K}\text{ and }{P}_{V}$ are shared projection matrices between several windows. We typically have $Q,K,V\in {\mathbb{R}}^{{M}^{2}\times d}$. Thus, the self-attention mechanism computes the attention matrix in a local window as

2 $$\text{Attention }\left(Q,K,V\right)={\text{SoftMax}}\left(\frac{Q{K}^{T}}{\sqrt{d}}+B\right)V,$$

where $B$ is the relevant positional encoding that can be learned. In practice, we concatenate the results for MSA by performing the attention function for h times in parallel, as described in Vaswani et al.^40^.

For more feature transformations, a multi-layer perceptron (MLP) with two fully connected layers and GELU non-linearity between them is utilized. The LayerNorm (LN) layer is introduced prior to both the MSA module and the MLP module. Additionally, a residual connection is used for both modules. Nevertheless, in the case when the partition is fixed for distinct layers, there exists a lack of interconnectivity among local windows. Hence, to facilitate cross-window connections, a combination of regular and shifted window partitioning techniques is used^45^. Specifically, shifted window partitioning involves moving the feature by $\left( {\left\lfloor \frac{M}{2} \right\rfloor ,\left\lfloor \frac{M}{2} \right\rfloor } \right)$ pixels prior to the partitioning process. The whole procedure is calculated per:

3 $$\begin{aligned} & \hat{X}^{l} = W - MSA\left( {LN\left( {X^{{l - 1}} } \right)} \right) + X^{{l - 1}} \\ & X^{l} = MLP\left( {LN\left( {\hat{X}^{l} } \right)} \right) + \hat{X}^{l} \\ & \hat{X}^{{l + 1}} = SW - MSA\left( {LN\left( {X^{l} } \right)} \right) + X^{l} \\ & X^{{l + 1}} = MLP\left( {LN\left( {\hat{X}^{{l + 1}} } \right)} \right) + \hat{X}^{{l + 1}} \\ \end{aligned}$$

#### Regional Feature Extraction Block (RFEB)

**Figure 6:**
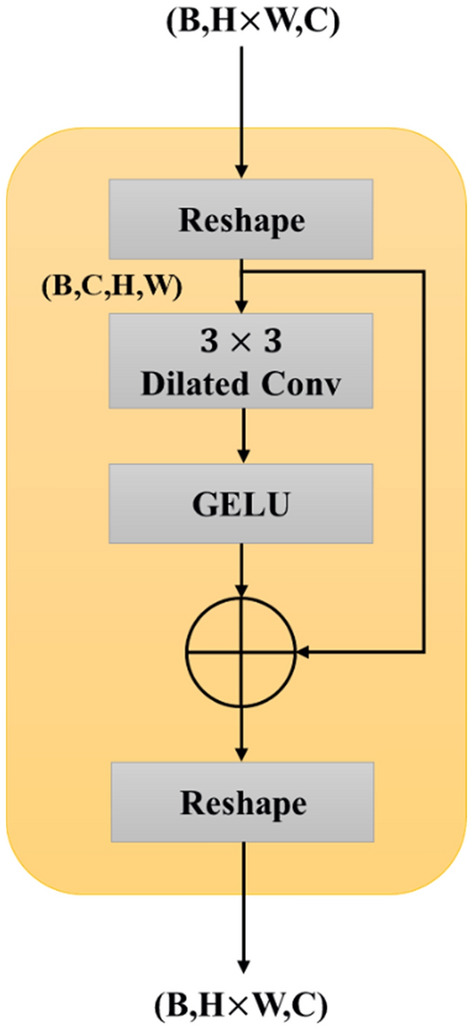
Structure of regional feature extraction block.

The detection of local correlation and structural information may be compromised by position encoding in a transformer. The Swin Transformer incorporates a shift window scheme; however, it fails to adequately encode a significant amount of spatial context information. To tackle this issue, a proposed solution is the implementation of a regional feature extraction block (RFEB), which is positioned prior to the Swin Transformer block, as depicted in Fig. 6.

The RFEB first performs a conversion process wherein a set of vector features is transformed into a spatial feature map. This conversion is necessary since the Swin Transformer model replaces the typical CNNs' feature maps with vectors. Consider the conversion of a token with the dimensions $(B, H \times W, C)$ into a feature map with the dimensions $(B, C, H, W)$ as an example. This is followed by the addition of a $3 \times 3$ layer dilated convolutions^55^ (dilation = 2) and a GELU activation function, and the inclusion of a residual connection to boost the spatial local feature extraction while maintaining a sizeable receptive field. The feature map is then given to the Swin Transformer block after being reshaped to $(B, H \times W, C)$. Dilated convolution's properties expand the spatial image's receptive field, allowing for the effective coding of a wide variety of contextual information at various scales. Dilated convolution provides the receptive field's expansion. Unlike traditional 3 × 3 convolutions, dilated convolutions with the same kernel size have a 7 × 7 receptive field, allowing for feature resolution enhancement without sacrificing field size.

### Loss computation

**Figure 7:**
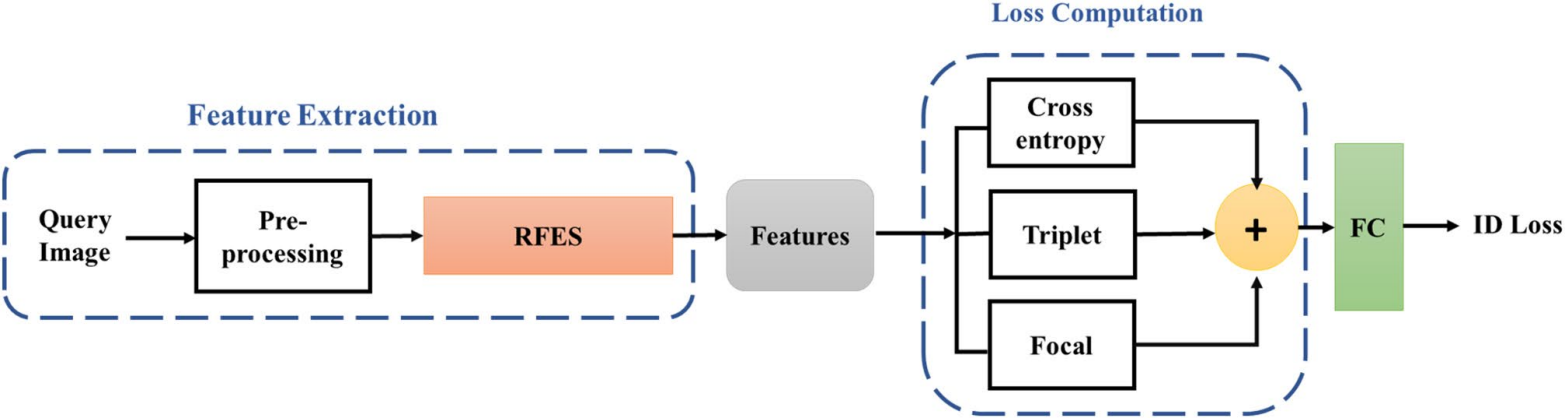
Fusion loss computation.

Following the feature generation phase, the resultant features are sent to the fusion loss stage, where three distinct loss functions, namely cross-entropy, triplet loss, and focal loss, are calculated. The results are then sent to a fully connected (FC) layer for ID prediction. Figure 7 illustrates the presented loss computation.

Each loss function focuses on different aspects of the learning task. The cross-entropy loss encourages correct classification, the triplet loss focuses on inter-sample similarities, and the focal loss addresses class imbalance issues. The proposed model learns not only to accurately classify but also to capture fine-grained similarities and effectively handle hard and imbalanced data, which increases the model's discriminative strength and facilitates the model's ability to learn effectively.

**Cross-entropy loss** Following is the definition of the cross-entropy loss function for many classes:

4 $${L}_{CE}\left({p}_{i},{y}_{i}\right)=-{y}_{i}{\text{log}}{p}_{i}$$

${p}_{i}$ indicates the probability obtained by the *i*-th sample's predicted person classification score, and ${y}_{i}$ represents the true label of the *i*-th sample.

**Triplet loss** In terms of metric learning, triplet loss is the most popular. Numerous metric learning techniques have been developed to enhance the performance of the triplet loss. One of the benefits of using the triple loss approach is its ability to facilitate the acquisition of intricate image details throughout the learning process. Each iteration involves the input of three paired images: an anchor picture $a$, a positive sample $p$ with the same ID as $a$, and a negative sample $n$ with a different ID. The mathematical expression for the triplet loss function is given by:

5 $${L}_{T}=max\left({d}_{a,p}-{d}_{a,n}+m{\text{arg}}in,0\right)$$

The Euclidean distance, ${d}_{a,p}$, is determined by the feature vectors of $a$ and $p$, and ${d}_{a,n}$, in a similar manner.

**Focal loss** The expression for the Focal loss function in the context of many categories is given by:

6 $${L}_{F}=\sum_{i=1}^{n} {\left(1-{p}_{i}\right)}^{\gamma }{L}_{CE}\left({p}_{i},{y}_{i}\right)$$

$n$ is the number of categories, whereas $\gamma$ is a hyperparameter that has a value larger than zero. The phrase ${\left(1-{p}_{i}\right)}^{\gamma }$ is used to increase the weight of the loss of the hard-to-separate samples in the overall loss and decrease the influence of the easy-to-separate samples. Due to the increased loss of hard-to-separate samples during training, the model is more attentive to these samples. It addresses the issue of a high number of easy-to-separate samples, reducing the total loss and enhancing the model's capacity for judgment regarding hard-to-separate samples.

The fusion loss uses a combination of cross-entropy loss, triplet loss, and focal loss. The use of cross-entropy loss promotes accurate classification. The triplet loss function is used to group data together in the feature space and get knowledge about the similarity between these samples. Moreover, Focal loss classifies the samples in the feature space by learning the interface of different feature space samples. The goal of using fusion loss is to improve the network by letting different loss functions limit each other. This aids network learning of representative characteristics. The fusion loss is expressed as:

7 $${L}_{Fusion}={L}_{CE}+{{L}_{T}+L}_{F}$$

### Re-ranking optimization

**Figure 8:**
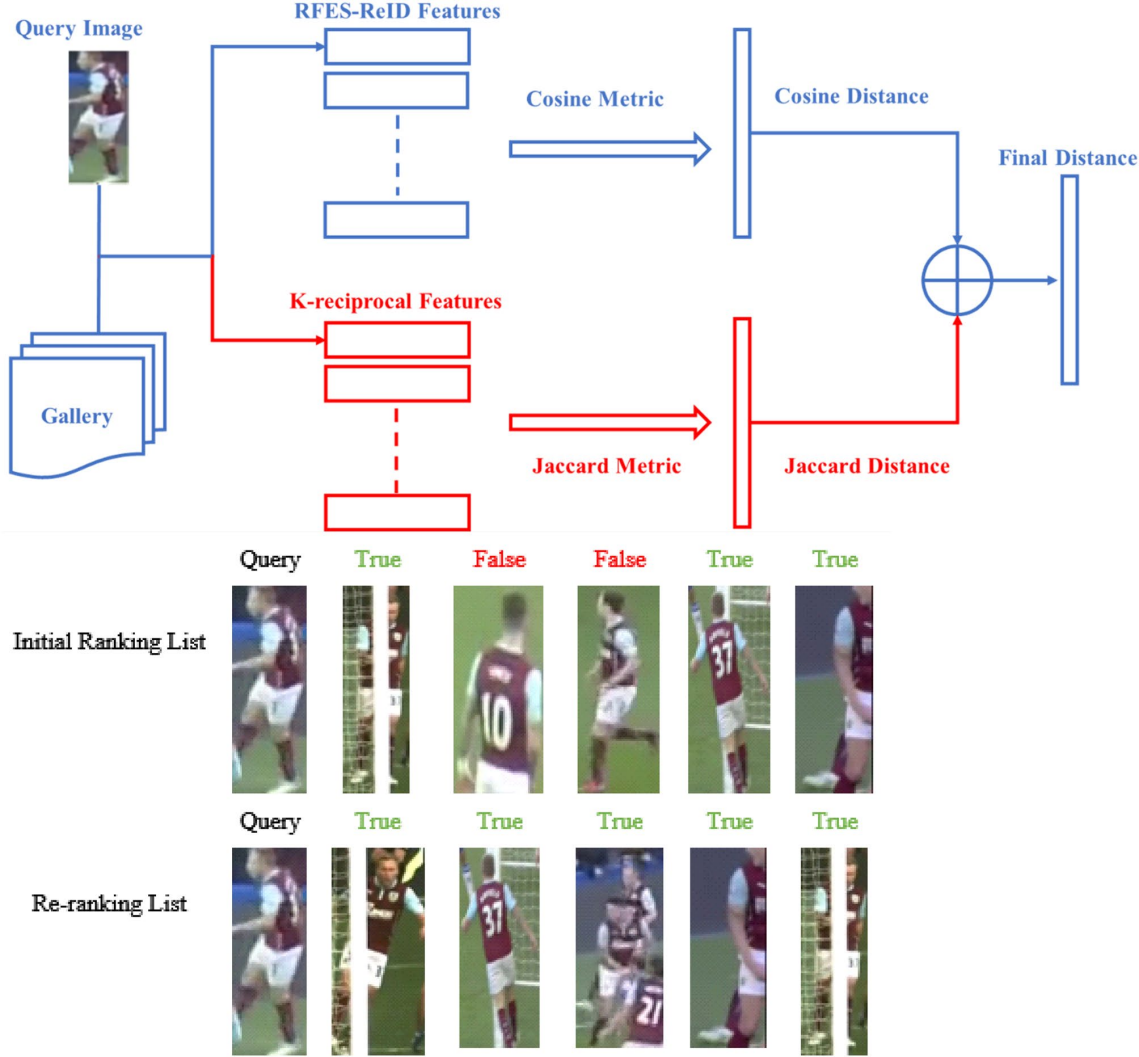
Re-ranking procedure for Player Re-ID.

In the inference stage of the suggested method, re-ranking optimization is used to improve the accuracy of the final prediction of person ReID. Figure 8 shows that re-ranking with k-reciprocal encoding^56^, which is a post-processing method, is done after the first list of IDs has been obtained. The proposed method uses re-ranking to improve prediction accuracy while re-identifying soccer players. In our implementation the same parameters are used as in the original paper^56^. Once the first ranked list is obtained, the top-k samples from this list are encoded as reciprocal neighbor features. These features are then leveraged to get k-reciprocal features. The Jaccard distance is assessed after the k-reciprocal features of both images have been identified. The final distance is then calculated by averaging the Jaccard distance with the Manhalanobis distance of feature appearance. The initial ranking list is then updated based on the final distance.

## Experimental results and performance evaluation

### Datasets and settings

Market-1501 and SoccerNet-V3 Re-identification are two benchmark ReID datasets that we used in our experiments. The following provides brief explanations of these datasets:

Market-1501^18^ contains 32,668 pedestrian images that were gathered by six campus cameras. It is separated into two groups. There are 12,963 images of 751 different IDs in the training set. The testing set also includes 19,281 images of 750 different IDs.

SoccerNet-v3 ReID^19,57^ were used for additional evaluation of our experiments. This dataset consists of 340,993 player thumbnails and images from their replays that were taken from SoccerNet videos of various events. The data is split into the train, validation, test, and challenge, respectively. There are a total of 248,234 samples in the training data. There are 34,989 gallery images and 11,777 query images in the test split. On the other hand, according to the challenge website^19^, player identity labels are created from linkages between bounding boxes inside an action and are thus only valid within the specified action. Since player ID tags do not stay the same from one action to another, a assigned player has a different ID for every action they are in. Because of this, only samples from the same activity are compared to one another throughout the assessment process. Therefore, we just need to compare each query sample to the gallery examples that have the same action. We exclusively train our networks on the train split to evaluate them on the test split.

**Evaluation metrics** To evaluate the performance of the ReID technique, two widely used evaluation metrics, namely Cumulative Matching Characteristic (CMC) and Mean Average Precision (mAP), are utilized. The CMC metric considers ReID as a ranking issue. Therefore, we focus on reporting the cumulative matching accuracy at Rank-1. Rank-1 is the standard accuracy at which the model generates the input identity with the greatest probability. According to Zheng et al.^18^, the mean average precision (mAP) considers ReID as an object retrieval issue.

### Experimental settings

The main challenges noted in our analysis are sample imbalance and lack of robustness. This lack of robustness refers to the system's vulnerability to variations or changes in input data, particularly when dealing with multiple-input resolutions, and it can impact the system's ability to maintain consistent performance across different situations. To overcome this issue, a pre-processing phase is implemented in which IDs in the SoccerNet-v3 dataset's training set with less than four images are removed. During the training process, the images used for training are subjected to various augmentation techniques, such as random horizontal flipping, random cropping, and random erasing^58^. These techniques are used with the aim of enhancing the robustness of the model. The training parameters have been taken from the Swin Transformer paper's settings. The batch size is configured as 32, comprising 8 unique IDs, with each ID encapsulating 4 images. The number of windows is determined by dividing the original article into a grid of $4\times 4$. The input images have a size of $224\times 224$. AdamW^59^ optimizer is employed for 120 epochs with a cosine decay learning rate scheduler and 10 epochs of linear warm-up. The learning rate is initialized as 0.001, and a weight decay of 0.05 is used. The margin of the triple loss is set at 0.3. The experimental running environment is the Windows 11 Home operating system. The processor is an Intel 13th Gen Core i9-13900KF, the memory is 64GB, the graphics processing card is an Nvidia GeForce RTX 4080 (16 GB). Also, the Cuda, Python, and Pytorch versions are 11.3, 3.6, and 1.10.4, respectively.

### Ablation studies

Table 1 lists the results of ablation research on individual components of the proposed method. The components include the backbone (Swin-T) and the RFEB, evaluated on both the Market-1501 and SoccerNet-v3 datasets. Starting with the backbone, incorporating the RFEB without fusion loss and re-ranking leads to improved performance in terms of Rank-1 accuracy and mAP. Introducing the fusion loss without re-ranking further enhances the results, demonstrating the significance of incorporating this component. However, the highest performance is achieved when both the fusion loss and re-ranking are combined, resulting in the highest Rank-1 accuracy and mAP values across both datasets. These findings emphasize the importance of the RFEB, fusion loss, and re-ranking in optimizing the proposed method, showcasing their collective impact on the accuracy and precision of person ReID in the Market-1501 and SoccerNet-v3 datasets.

**Table 1:** Ablation testing of individual components: impact on the proposed method's performance.

	Methods		Market-1501		SoccorNet-v3	
	Fusion loss	Re-ranking	Rank-1 (%)	mAP (%)	Rank-1(%)	mAP (%)
Backbone (Swin-T)	–	–	94.15	83.00	75.34	80.16
+ RFEB	–	–	94.73	83.54	79.63	81.39
	✓	–	95.7	87.3	81.8	85.0
	✓	✓	96.2	89.1	84.1	86.7

### The impact on fusion loss function

Table 2 examines the impact of different loss functions on the performance of RFES-ReID, with a particular focus on the fusion loss function. Results show that the fusion loss consistently outperforms other loss functions across both datasets, Market-1501 and SoccerNet-v3. RFES-ReID utilizing the fusion loss achieves impressive Rank-1 accuracies of 95.10% on Market-1501 and 81.82% on SoccerNet-v3, along with the mAP values of 86.97% and 85.02%, respectively. These findings highlight the significant impact of the Fusion loss function in enhancing the accuracy and precision of RFES-ReID. By effectively combining multiple loss components, the fusion loss enables the model to better capture and discriminate person features, leading to superior performance compared to other loss functions such as Cross-entropy, Triplet, and Focal. The results underscore the importance of incorporating the fusion loss function in RFES-ReID for achieving improved person ReID outcomes.

**Table 2:** The effectiveness of loss selections on RFES-ReID.

Methods	Market-1501		SoccorNet-v3	
	Rank-1 (%)	mAP (%)	Rank-1 (%)	mAP (%)
Cross-entropy	92.0	79.31	74.63	78.96
Triplet	94.73	83.54	79.63	81.39
Focal	94.89	86.78	80.06	84.25
Cross-entropy + Triplet	94.86	84.23	79.87	82.54
Cross-entropy + Focal	94.96	86.81	80.45	84.58
Triplet + Focal	95.23	87.04	80.92	84.71
Fusion	95.7	87.3	81.8	85.0

**Figure 9:**
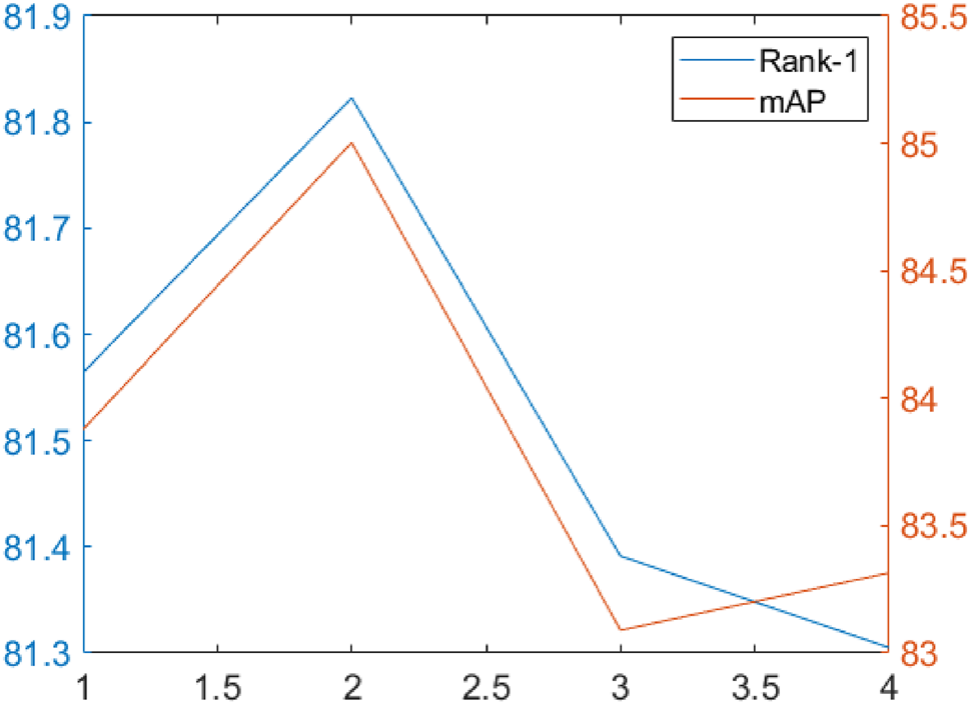
The impact of different $\gamma$ on Rank-1 (%) and mAP on SoccerNet-v3 was used to choose $\gamma$.

The proposed model uses fusion loss, including a combination of cross-entropy loss, triplet loss, and focal loss. The cross-entropy loss promotes correct classification. On the other hand, the triplet loss focuses on inter-sample similarities. The focal loss increases the weighting of the loss associated with hard-to-separate samples within the overall loss function by using the term ${\left(1-{p}_{i}\right)}^{\gamma }$, while simultaneously decreasing the weighting of the loss associated with easily-to-separate data. During the training process, the amplification of loss for hard-to-separate data leads to increased attention from the model toward these samples. The SoccerNet-v3 dataset is used to evaluate a significant hyperparameter, and afterwards, the optimal hyperparameter is chosen. The results of the experiment conducted on SoccerNet-v3 are shown in Fig. 9. It has been shown that the relation between mAP, Rank-1, and $\gamma$ is not linear and that it does not become better as $\gamma$ increases. The best result is achieved when $\gamma =2$, hence the focal loss parameter $\gamma =2$ is taken into consideration.

### Comparison with the state-of-the-art method

The proposed method is compared with other methods on Market-1501 and SoccerNet-v3 datasets. The methods utilize various backbones and input sizes, with evaluation metrics including Rank-1 accuracy and mAP. Table 3 presents a comparison of different methods on the Market-1501 dataset, which is commonly used for person ReID research. Among them, PCB^6^, ABDNet^60^, SAN^61^, PGFA^62^, MGN^7^, and RGA-SC^63^ are based on CNN methods and rest is based on transformer methods. On the Market-1501, the proposed method outperforms CNN-based methods, indicating that the transformer-based method outperformes the CNN-based method, and the use of transformers to solve the problem of person ReID is becoming more common and unavoidable. We also surpass transformer-based method except TransReID-SSL^64^. But as it is clear from Table 3 they provide competitive results. The proposed method stands out with the Rank-1 accuracy of 96.2% and the mAP of 89.1%. These results demonstrate the effectiveness of the RFES-ReID method in accurately identifying and matching persons in the Market-1501 dataset, positioning it among the state-of-the-art techniques for person ReID tasks.

**Table 3:** Comparison with the state-of-the-art methods on Market-1501 dataset.

Methods	Backbone	Size	Rank-1 (%)	mAP (%)
ResNet50	CNN	256 × 128	88.8	71.5
OSNet		256 × 128	94.8	84.9
PCB^6^		384 × 128	93.8	81.6
ABDNet^60^		384 × 128	95.6	88.3
SAN^61^		256 × 128	96.1	88.0
PGFA^62^		256 × 128	91.2	76.8
MGN^7^		384 × 128	95.7	86.9
RGA-SC^63^		256 × 128	96.1	88.4
Baseline	ViT-B/16	256 × 128	94.7	86.8
TransReID^43^		256 × 128	95.0	88.2
TransReID-SSL^64^		256 × 128	**96.2**	**91.3**
Baseline	DeiT-B/16	256 × 128	94.4	86.6
TransReID^43^		256 × 128	94.7	88.0
Baseline	Swin-T	224 × 224	94.1	86.0
TL-TransNet + BAR^46^		224 × 224	95.34	92.60
Ours (w/o re-ranking)		224 × 224	95.7	87.3
Ours (with re-ranking)		224 × 224	**96.2**	89.1

Significant values are in bold.

Table 4 provides a comparison of various methods applied to the SoccerNet-v3. The methods utilize different backbones, such as CNN, ViT, DeiT, and Swin-T, and have varying input sizes. Notable observations include the CNN-based methods with the lowest performance, while transformer-based methods show higher accuracies. The TransReID-SSL method stands out with an impressive Rank-1 accuracy of 83.8% and the mAP of 80.1%. However, the proposed method (RFES-ReID), utilizing the Swin-T backbone, performs exceptionally well with an 84.1% Rank-1 accuracy and the mAP of 86.7%. Overall, these results showcase the advancements made in video-based person ReID techniques for soccer-related applications.

**Table 4:** Comparsion with the state-of-the-art methods on SoccerNet-v3 dataset.

Methods	Backbone	Size	Rank-1 (%)	mAP (%)
ResNet50	CNN	256 × 128	48.4	59.1
OSNet		256 × 128	69.2	76.4
Baseline	ViT-B/16	256 × 128	68.2	75.7
TransReID^43^	ViT-B/16	256 × 128	68.6	77.2
TransReID-SSL^44^	ViT/S-16	256 × 128	83.8	80.1
Sports Re-ID^65^	ViT/S-16	256 × 128	81.5	86.0
Baseline	DeiT-B/16	256 × 128	65.0	73.2
TransReID^43^		256 × 128	65.4	74.6
Baseline	Swin-T	224 × 224	75.3	80.2
Ours (w/o re-ranking)		224 × 224	81.8	85.0
Ours (with re-ranking)		224 × 224	**84.1**	**86.7**

Significant values are in bold.

Tables 3 and 4 describe the effect of re-ranking on the RFES-ReID method for Market-1501 and SoccerNet-v3. Without applying re-ranking, RFES-ReID achieves the Rank-1 accuracy of 95.7% and the mAP of 87.3% on Market-1501, while on SoccerNet-v3, it attains the Rank-1 accuracy of 81.8% and the mAP of 85.0%. Meanwhile, the application of re-ranking leads to notable improvements in performance. By applying re-ranking, RFES-ReID achieves an increased Rank-1 accuracy of 96.2% on Market-1501 (an increase of 0.5%) and 84.1% on SoccerNet-v3 (an increase of 2.3%). Additionally, the mAP also shows improvement, reaching 89.1% on Market-1501 (an increase of 1.8%) and 86.7% on SoccerNet-v3 (an increase of 1.7%). These results highlight the positive impact of re-ranking on the RFES-ReID method, resulting in enhanced accuracy and precision in identifying and matching persons in both datasets.

This paper introduces an innovative approach to person and soccer player ReID by utilizing the Swin Transformer as the backbone network, addressing the shortcomings associated with traditional CNNs and their computational demands. The proposed RFES method improves feature extraction precision for smaller objects and enhances the model's local perception capabilities by incorporating the strengths of CNNs. Additionally, enhancements in the soccer player ReID network are achieved through the integration of cross-entropy loss, triplet loss, and focal loss, facilitating precise classification, handling of imbalanced data, and consideration of inter-sample similarities and challenging-to-distinguish samples.

The RFES-ReID framework demonstrates competitive performance across person and soccer player ReID benchmarks, specifically Market-1501 and SoccerNet-v3. The proposed method consistently outperforms CNN-based approaches on the Market-1501 dataset. Moreover, in comparison to various methods applied to SoccerNet-v3, the RFES-ReID method exhibits superior accuracy. Notably, the TransReID-SSL method shows promising results, but the RFES-ReID method, leveraging the Swin-T backbone, performs exceptionally well in terms of both Rank-1 accuracy and mAP, solidifying its position among the state-of-the-art techniques for person ReID tasks and surpassing CNN-based methods.

### Discussion on comparative performance

This paper introduces an innovative approach to soccer player and person ReID by utilizing the Swin Transformer as the backbone network, addressing the shortcomings associated with traditional CNNs and their computational demands. The proposed RFES method improves feature extraction precision for smaller objects and enhances the model's local perception capabilities by incorporating the strengths of CNNs. Additionally, enhancements in the soccer player ReID network are achieved through the integration of cross-entropy loss, triplet loss, and focal loss, facilitating precise classification, handling of imbalanced data, and consideration of inter-sample similarities and challenging-to-distinguish samples.

The RFES-ReID framework demonstrates competitive performance across person and soccer player ReID benchmarks, specifically Market-1501 and SoccerNet-v3. The proposed method consistently outperforms CNN-based approaches on the Market-1501 dataset. Moreover, in comparison to various methods applied to SoccerNet-v3, the RFES-ReID method exhibits superior accuracy. Notably, the TransReID-SSL method shows promising results, but the RFES-ReID method, leveraging the Swin-T backbone, performs exceptionally well in terms of both Rank-1 accuracy and mAP, solidifying its position among the state-of-the-art techniques for person ReID tasks and surpassing CNN-based methods.

## Conclusions

There are some important distinctions between surveillance ReID applications and player ReID in broadcast video. Strong features in the images are required because of these distinctions. Due to data availability, we chose to concentrate on soccer in this study, although the concepts covered here are relevant to numerous team sports. This paper proposed a Regional Feature Extraction Swin Transformer (RFES) to address the soccer player ReID problem. Firstly, the Swin Transformer is used as a feature extraction network to get around both the long-range dependencies issue of conventional CNNs and the high computational complexity of transformers. Secondly, a regional feature extraction module is applied to extract low-dimensional feature representations. Finally, we integrate three different loss functions to manage unbalanced data, highlight hard situations, and pay more attention to hard-to-separate samples. The rank list's quality was then raised by using re-ranking with k-reciprocal encoding. The results of the experiment on the Market-1501 and SoccerNet-v3 datasets show that the suggested model outperforms state-of-the-art approaches while being straightforward and efficient. For future study, we will use the provided model to extract more effective features and improve additional team sports player ReID tasks by considering perspective difference.

## Data Availability

The datasets analyzed during the current study are available in the following public domain resources: https://www.soccer-net.org/data, https://zheng-lab.cecs.anu.edu.au/Project/project_reid.html.

## Code availability

The utilized codes and data are available on request to enable the method proposed in the manuscript to be replicated by readers.
